# The role of some metal ions in enhancement of photocatalytic activity of Fe_2_O_3_–V_2_O_5_ binary oxide

**DOI:** 10.3906/kim-2008-57

**Published:** 2021-04-28

**Authors:** Eda SINIRTAŞ İLKME, Gülin Selda POZAN SOYLU

**Affiliations:** 1 Department of Chemical Engineering, Engineering Faculty, İstanbul University-Cerrahpaşa, İstanbul Turkey

**Keywords:** 2,4-dichlorophenol, degradation, photocatalyst, mixed oxide, UV irradiation

## Abstract

Fe_2_O_3_-V_2_O_5_ mixed oxides were synthesized with solid-state dispersion (SSD) and coprecipitation methods. In addition, transition metal oxides such as CuO, NiO, and CO_3_O_4_ were successfully loaded on the synthesized catalyst (Fe_2_O_3_-V_2_O_5_) using the SSD method. The composite catalysts were inspected for their photocatalytic activities in degrading 2,4-dichlorophenol under UV light enforcement. The produced samples were analyzed using X-ray diffraction, X-ray photoelectron spectroscopy, Fourier transform infrared spectroscopy, diffuse reflectance spectroscopy, scanning electron microscopy, photoluminescence, and the Brunauer–Emmett–Teller method. The addition of transition metal oxides improved the photocatalytic activity of Fe_2_O_3_-V_2_O_5_ (SSD). 1CuO wt% Fe_2_O_3_-V_2_O_5_ exhibited the highest percentage of 2,4-dichlorophenol degradation (100%) and the highest reaction rate (1.83 mg/L min) in 30 min. This finding is attributed to the distribution of CuO.

## 1. Introduction

Chlorophenol compounds and their derivatives are highly toxic compounds [1–5]. Due to their adverse effects, US Environmental Protection Agency (EPA) [6] and European regulatory authorities [7] have listed chlorophenols as significant toxins. The removal of these hazardous organic pollutants has become critical to build up eco-friendly and economic techniques.

Heterogeneous photocatalytic oxidation has received substantial attention as a potentially competent method for breaking down refractory environmental pollutants. Recently, many studies have focused on doping with transition metal ions [8,9] and combining them with various narrow band gap semiconductors acting as photocatalysts [10,11]. CeO_2_, SnO_2_, ZrO_2_, Sm_2_O_3_, Fe_2_O_3_, TiO_2_, V_2_O_5_, WO_3_, ZnO, CdS, Bi_2_O_3_, Sb_2_O_3_, Al_2_O_3_, MoO_3_, and many others have been used as conventional photocatalyst due to their appropriate band gap, high photocatalytic activity, and chemical steadiness [12,13]. Some studies have shown that a large number of binary composites such as V_2_O_5_–TiO_2_ [14], V_2_O_5_–ZnO [15], Au/Fe_2_O_3_ [16], and BiVO_4_/FeVO_4_ [17], and a small number of ternary nanocomposites such as V_2_O_5_–WO_3_/TiO_2_ [18], Ni/MgO–ZrO_2_ [19], and N–Fe_2_O_3_/FeVO_4_ [20] have been designed and synthesized for organic pollution under visible illumination. Semiconductor particles that contain metal play a significant role in the exclusion of a set of ecological pollutants like phenols. Many records show that the addition of Fe^3+^, V, Ni, Pt, Cr^3+^, Cu^2+^, Co, Au, Pd, or other cations into particle can increase the photoactivity [21–29].

In nanotechnology, V_2_O_5_ and Fe_2_O_3_ are two well-known semiconductors for various photocatalytic applications. Iron oxide has been used due to its stability, narrow band gap, high efficiency, and environmentally friendly, nontoxic, and inexpensive nature [30,31]. Additionally, nanostructure vanadium pentoxide (V_2_O_5_) has been considered an important material due to its low cost, biocompatibility, availability, easy synthesis, and good electrical and optical properties. In the literature, there are studies with several applications such as catalysts, solar cells, as a cathode material in rechargeable lithium batteries, gas sensors, electrochromic devices, and electrooptic switches [32–38]. In addition, the use of mixed oxides in many technological fields, including lithium secondary batteries, methanation, and gas sensors, is an attractive way to produce materials with superior properties than the single components. Binary metal oxides have been widely used as photocatalysts for decades because the morphological properties of the individual oxides can be changed by formation of new sites in the interface between the components, or by incorporation of one oxide into the lattice of the other. This enhancement was attributed to gradually increasing shift of the conduction bands with increasing metal oxide contents, resulting in a stronger reduction power of photogenerated electrons and promoting the improved photocatalytic activity.

The activity of metal oxide catalysts can be enhanced by addition of second and third metal oxides. Each components of the ternary system MO–V_2_O_5_–Fe_2_O_3_ and individual oxides existing in this lateral system could easily catalyze a series of chemical reactions [39–41].

In the present work, binary metal oxide catalysts Fe_2_O_3_ – V_2_O_5_ (FeV-Pure) were produced with the solid-state dispersion (SSD) method and the effect of transition metal oxides such as CuO, NiO, and CO_3_O_4_ additives were examined on the photocatalytic efficiency. The photocatalytic activity of the catalysts was examined for the degradation of organic pollutant 2,4-dichlorophenol (2,4-DCP) under UV radiation. The catalysts were studied with X-ray diffraction (XRD), X-ray photoelectron spectroscopy (XPS), diffuse reflectance spectroscopy (DRS), and scanning electron microscopy (SEM). Fourier transformation infrared (FT-IR), Braun–Emmet–Teller (BET) were used for analyzing the surface area and the relationship between the structure of the catalysts and photocatalytic activities was evaluated. 

## 2. Materials and methods

### 2.1. Materials

Starting ingredients used for preparation of the catalysts were Cu(NO_3_)2.3H_2_O, Ni(NO_3_)2.6H_2_O, Co(NO_3_)2.6H_2_O (reagent grade, Sigma-Aldrich, Darmstadt-Germany), V_2_O_5_ (Vanadium(V)-oxide, MERCK, Darmstadt-Germany, Fe_2_O_3_ (Iron(III)-oxide, MERCK), ethanol (absolute), and P25 (consisting of 75% anatase and 25% rutile with a speciﬁc BET-surface area of 50 m^2^/g and primary particle size of 20 nm). The organic compounds used in the photocatalytic studies were 2,4-DCP, 4-chlorophenol, 2-chlorophenol, phenol, hydroquinone, catechol, and methanol (for HPLC, ≥99 %) and they were purchased from Fluka Company (Germany) and used without further purification. Deionized (D.I.) water was used for the preparation of all the catalysts and for the dilution of the 2,4-DCP solution.

### 2.2. Catalyst preparation

 FeV-Pure catalyst was prepared with the SSD method. Metal oxide and FeV-Pure were mixed thoroughly using ethanol as a solvent using an agate mortar and pestle. The solvent was then removed by evaporation whilst mixing the sample. The resulting product was then left to dry at 110 °C and calcined at 450 °C for 6 h to attain binary oxide catalysts. The binary oxide was grounded at a steady vibration rate of 300 rpm for 15 min in a Retsch MM 200 vibrant-ball mill by 12mm ZrO_2_ milling ball in ZrO_2_ milling container. In this method, V_2_O_5_ and Fe_2_O_3_ samples were mixed in the specific proportion of 1:1. 

Furthermore, FeV-Pure (CP) catalyst was prepared with the coprecipitation (CP) method. In this method, Fe (NO_3_)_3_.9H_2_O and NH_4_VO_3_ were dissolved in deionized water separately and then the solutions were mixed. The mixture was stirred for 1 h in a magnetic stirrer. Afterward, the samples were left to dry at 50 °C for 15 h and calcined at 500 °C for 4 h to obtain the ternary oxide catalyst.

The pure metal oxides such as CuO, NiO, and CO_3_O_4_ were prepared with the CP method. Cu(NO_3_)_2_.3H_2_O, Ni(NO_3_)_2_.6H_2_O, and Co(NO_3_)_2_.6H_2_O were dissolved in deionized hot water and the solution was heated up to 65 °C. This solution mixture was precipitated by gradual addition of NH3 solution (25 wt %) till pH reached a value of 10. The resulting solution was gently stirred for 2 h at 65 °C. Following that, the solution was placed under 500 W microwave irradiation for about 3 min. The product obtained was then ﬁltered and washed several times with deionized water and left to dry at 100 °C for about 20 h followed by calcination at 500 °C for 5 h.

The metal-oxide-loaded FeV-Pure catalyst was prepared with the SSD method. SSD initially involves mixing of metal oxide and FeV-Pure thoroughly using ethanol in in an agate mortar, the solvent was then removed by evaporation while mixing. Samples prepared by this method were dried at 110 °C and calcined at 450 °C for 6 h to obtain binary oxide catalysts. The resultant binary oxide was grounded at a constant vibration rate of 300 rpm for 15 min in a Retsch MM 200 vibrant-ball mill by 12mm ZrO_2_ milling ball in ZrO_2_ milling container. Metal oxide loading of the catalysts was nominally 0.5, 1, 3, 5, and 7 wt% and reported as the weight percentage. For instance, 1CuO-(FeV-Pure) means that the catalyst contained nominally 1% CuO by weight.

### 2.3. Catalyst characterization

The surface area of the powder was determined with the BET method. Before the measurements, the samples were degassed at 200 °C for 3 h. X-ray powder diffraction patterns of samples were attained using a Rigaku D/Max-2200 diffractometer with the CuKα (l = 1.540) radiation. All powders were scanned ranging from 10 to 80 at a rate of 2o/min (in 2θ). The average crystallite sizes of the samples were determined using the Scherrer formula. The morphological evaluation, i.e. particle size and powder distribution, was carried out with SEM (JEOL/JSM-6335F). The determination of the metal oxide and OH groups of the samples were characterized with FT-IR spectroscopy (Perkin Elmer Precisely Spectrum One, PerkinElmer Inc., Waltham, MA ,USA). UV–vis diffuse reflectance spectra of the samples were carried out on an UV–vis spectrophotometer (Shimadzu UV-3600, Japan) by using BaSO_4_ as the reference.

### 2.4. Evaluation of photocatalytic activity

The photoactivity evaluation of the samples was performed at room temperature (298 K) and atmospheric pressure. For all the experiments, 100 mg catalyst was taken and dispersed in 50 mL of 2,4-DCP solution having initial concentration of 25 mg/L and pH of 5 in a magnetic stirring machine. The reaction was done with quartz batch photo-reactor of cylindrical shape. LUZCHEM LZC-5 photo-reactor system was used in all the experiments and 64 W UV-A lamp (Luzchem LZC-UVA) was used as a light source. UV-A lamp has the highest light intensity at 312 nm and distance of illumination of 18 cm from the target. The light intensity of UV lamp used for degradation experiments was documented with an UV/visible power-meter (Smart Sensor-AR823). The photo-reactor system had a magnetic stirrer. Before the UV light was switched on, the solution was stirred for 60 min to make sure that the suitable adsorption equilibrium between the catalyst and the solution was reached. After irradiating the sample for about 2 h, the 2,4-DCP solution was filtered using a membrane filter (pore size 0.45 mm) and the filtrate was then used for total organic carbon (TOC) measurement with a TOC-V, Shimadzu equipment. 

The concentration of phenol and products were analyzed by HPLC (Thermo Finnigan, Canton, ABD) equipped with C-18 column. The mobile phase used in HPLC was a mixed solvent of methanol and water (70/30, v/v) with a flow rate of 1 mL/min.

## 3. Results and discussion

### 3.1. BET surface area

The surface areas of the samples were determined through adsorption of inert gas such as N2 for the purpose of observing the impact of metal addition and binary oxide structure on physical properties of the catalysts. The results were listed as given in Table. The surface area of pure Fe_2_O_3_ and V_2_O_5_ are 32 and 25 m2/g, respectively. Additionally, the comparison between the values of the surface area of FeV-Pure and metal-oxide-added FeV-Pure was made and the surface area value of bare catalyst was found to be higher than those of metal-oxide-added catalysts. Active metal plays a direct role in binary oxide catalysts in the formation of the physical structure; hence, the metal addition could lead to the formation of the surface area of the catalyst. For the FeV-Pure series, the surface areas decreased with the addition of metal oxides. This effect is due to the deposition of transition metal oxide particles on FeV-Pure surface and blocking the pores and channels. 

**Table:** TThe crystallite sizes, specific surface areas, band gap, morphology of materials and 2,4-DCP degradation efficiencies over 30 min (%)

Catalyst	Crystallitesize (nm)	SBET(m2 g–1)	Band gap(eV)	2,4-DCP degradationefficiencies over 30 min (%)	kr (mg/L min)
FeV-Pure (SSD)	44	10	2.09	56	1.04
FeV-Pure (CP)	43	-	2.48	48	0.52
1CO_3_O_4_ -(FeV-Pure)	33	5	2.07	87	1.20
1NiO-(FeV-Pure)	36	7	2.04	91	1.47
1CuO-(FeV-Pure)	32	8	1.96	100	1.83
V_2_O_5_	40	25	2.35	38	0.64
Fe_2_O_3_	58	32	2.03	43	0.78

### 3.2. X-ray diffraction analysis

**Figure 1:**
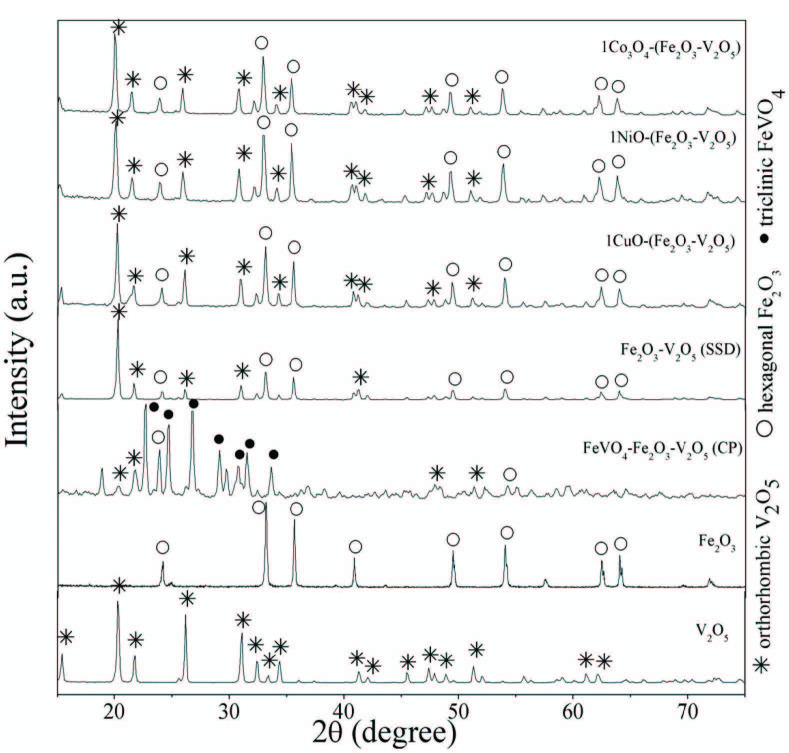
XRD patterns of V_2_O_5_,Fe_2_O_3_,Fe_2_O_3_-V_2_O_5_ (SSD), Fe_2_O_3_-V_2_O_5_ (CP), and metal-doped Fe_2_O_3_-V_2_O_5_ catalysts.

Diffraction patterns for pure Fe_2_O_3_, V_2_O_5_, synthesized FeV-Pure, and M-(FeV-Pure) (M= CuO, NiO, CO_3_O_4_) catalysts are shown in Figure 1. All related peaks were distinguished and recorded using the data accessed from the Joint Committee For Powder Diffraction studies (JCPDS). It was observed that all the catalysts were well crystallized. Referring to Figure 1, the X-ray diffraction patterns displayed that both orthorhombic V_2_O_5_ (JCPDS 41-1426), being the dominating phase, and hexagonal Fe_2_O_3_ (JCPDS-33-0664) were detected for FeV-Pure and M – (FeV-Pure) catalysts. XRD approved the existence of Fe_2_O_3_ and V_2_O_5_ in the mixed oxide, highlighting the successful entrance of Fe_2_O_3_ onto V_2_O_5_. For FeV-Pure and metal-oxide-doped catalysts, the dominant phase of V_2_O_5_ is orthorhombic when some portion of iron is buried into vanadium particles and the intensity peaks become more defined. Thus, crystallinity, the size of crystallite, and the phase structure of V_2_O_5_ have a significant function in photocatalytic activity. However, no noticeable peaks were found for the SSD of metal oxides such as CuO, NiO, and CO_3_O_4_ over FeV-Pure. This could be the result of the less amount of loading of metal oxide over FeV-Pure mixed oxides, which would indicate that the doped metal (CuO, NiO, and CO_3_O_4_) oxides were highly dispersed on the FeV-Pure support [42].

Additionally, FeV-Pure (CP) was also analyzed with an XRD to determine which chemicals are present. As shown in Figure 1, it can be seen that FeVO_4_ structure rises in the synthesis form of the CP technique. The creation indicates that the specific form and arrangement of the coprecipitation method have no effect in terms of photocatalytic activity. The synthesis technique of catalyst plays a significant role in the photocatalytic efficiency.

The Scherrer formula was used to calculate the mean crystallite sizes of the catalysts and the results are recorded in Table. According to the results, the crystallite sizes of samples were found as 32, 36, and 43 nm for 1CuO – (FeV-Pure), 1NiO – (FeV-Pure), and 1CO_3_O_4_ – FeV-Pure, respectively. The crystallite size of FeV-Pure decreased by addition transition metal oxides. The dissimilarity could result in differences in their structure and morphology [43].

### 3.3. Scanning electron microscopy 

**Figure 2:**
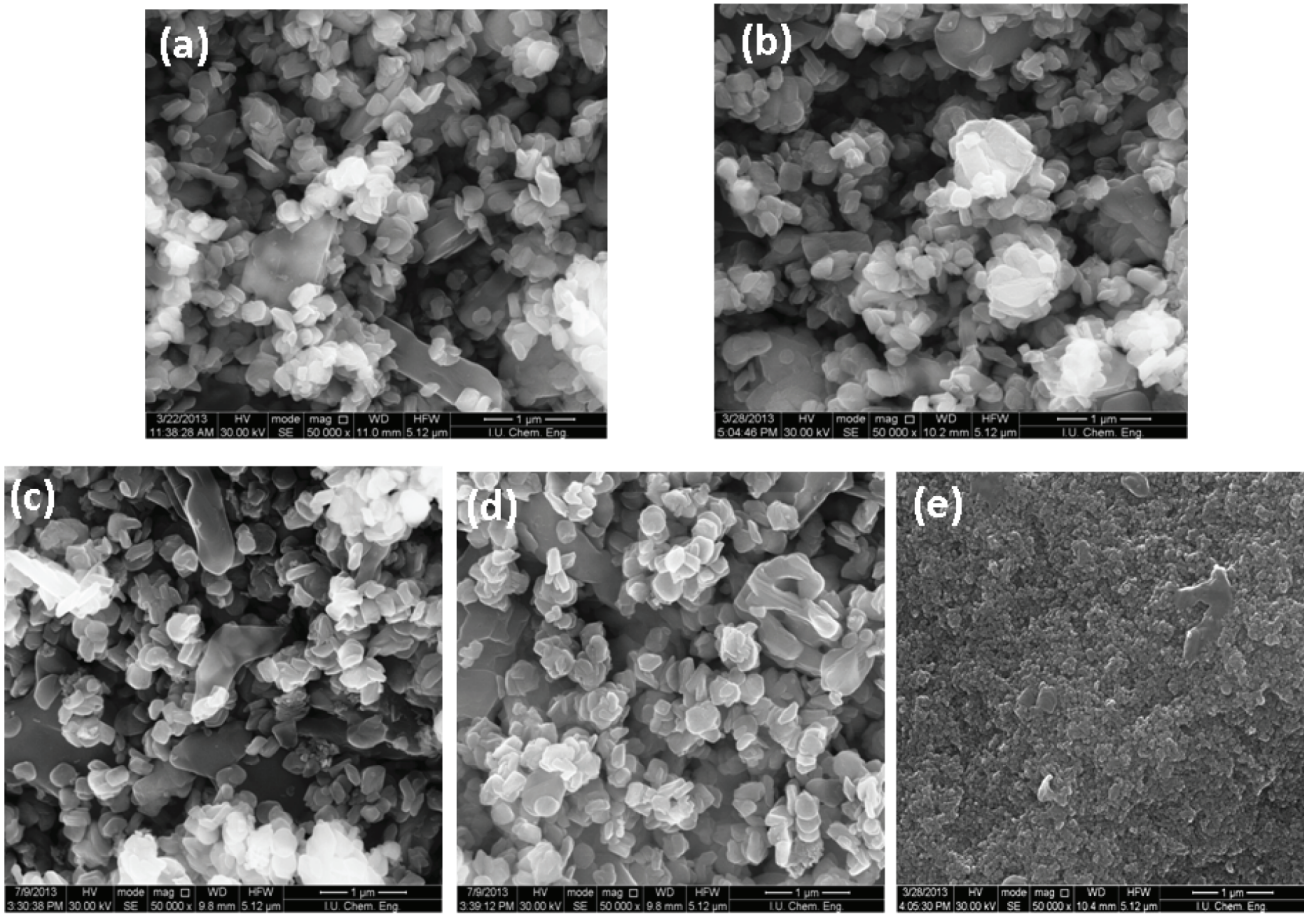
SEM images of (a) Fe2O3 – V2O5 (SSD), (b) 1CuO – (Fe2O3 – V2O5), (c) 1NiO – (Fe2O3 – V2O5), (d) 1Co3O4 – (Fe2O3 – V2O5), and (e) Fe2O3 – V2O5 (CP) catalysts.

Morphology of photocatalyts plays a key role in inﬂuencing the photocatalytic efficiency. The surface morphologies of FeV-Pure and FeV-Pure (CP) composite catalysts were seen clearly from SEM images displayed in Figure 2. According to the SEM images, FeV-Pure binary oxides present granular-like crystals. The surface morphology of FeV-Pure sample shows the spherical nanoparticles, micro- and macrograins attached to the catalyst surface. However, the morphology of the photo catalyst prepared through the CP technique differs from that of the SSD technique. As shown in Figure 2, it can be told that FeV-Pure (CP) was slightly agglomerated into nested shapes. The agglomeration can result in the decrease in the photocatalytic activity. In Figure 2, compared to the catalysts, the metal-oxide-doped catalysts also exhibited more regular and uniform spherical crystalline shape. CuO dispersion was seen on the entire surface of FeV-Pure in comparison to NiO and CO_3_O_4_. It has high photocatalytic efficiency due to the homogenously high dispersion on FeV-Pure. As can be observed, the metal-oxide-doped catalysts had a similar potential morphology. 

### 3.4. UV–vis diffuse reﬂectance spectroscopy

**Figure 3:**
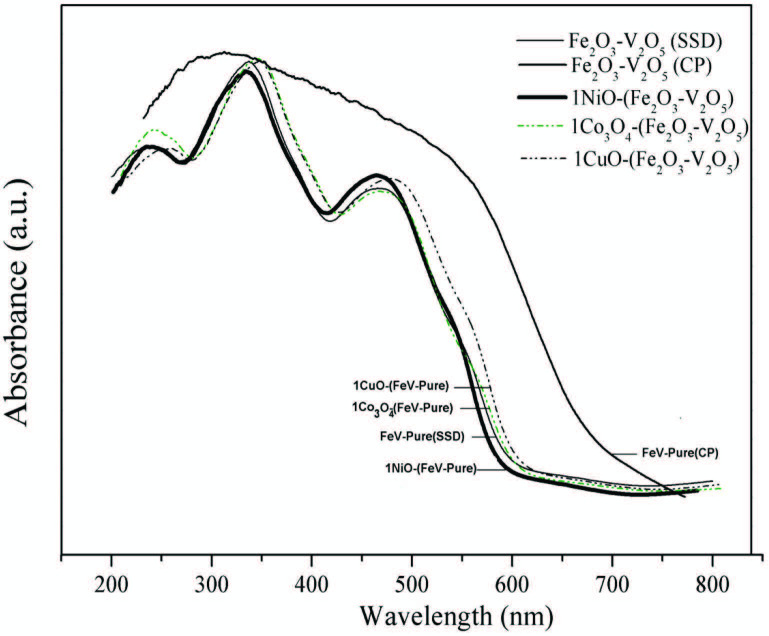
DRS spectra of V2O5, Fe2O3, Fe2O3 – V2O5 (SSD), Fe2O3 – V2O5 (CP) and metaldoped Fe2O3 – V2O5 catalysts.

The optical absorption characteristic relevant to the electronic structure feature acts as a main factor in defining the photocatalytic efficiency [44]. UV–vis diffuse reflectance spectra of the samples are shown in Figure 3. The catalysts showed strong absorption in visible-light region as well in the UV light region, which suggests the likelihood of high photocatalytic efficiency over these materials under visible-light radiation. The band gap energy (Eg) for all the catalysts were calculated according to the equation Eg = 1240/k (eV), where k is the absorption edge wavelength (nm), acquired from the intercept through the tangent of the absorption jump and X-axis, and are given in Table [45].

As a result of metal oxide doping, the band gap energy of FeV-Pure catalyst was found to be decreasing. Among all the synthesized catalysts, large decrease in the band gap value was seen for CuO-doped catalyst and the band gap was around 1.96 eV. The change of the band gap was ascribed to the charge flow from CuO to FeV-Pure layer causing downward shifting of the conduction band and upward shifting of the valence band. In addition, this change on the band gap energy positively affects the photocatalytic activity.

### 3.5. FT-IR spectroscopy 

**Figure 4:**
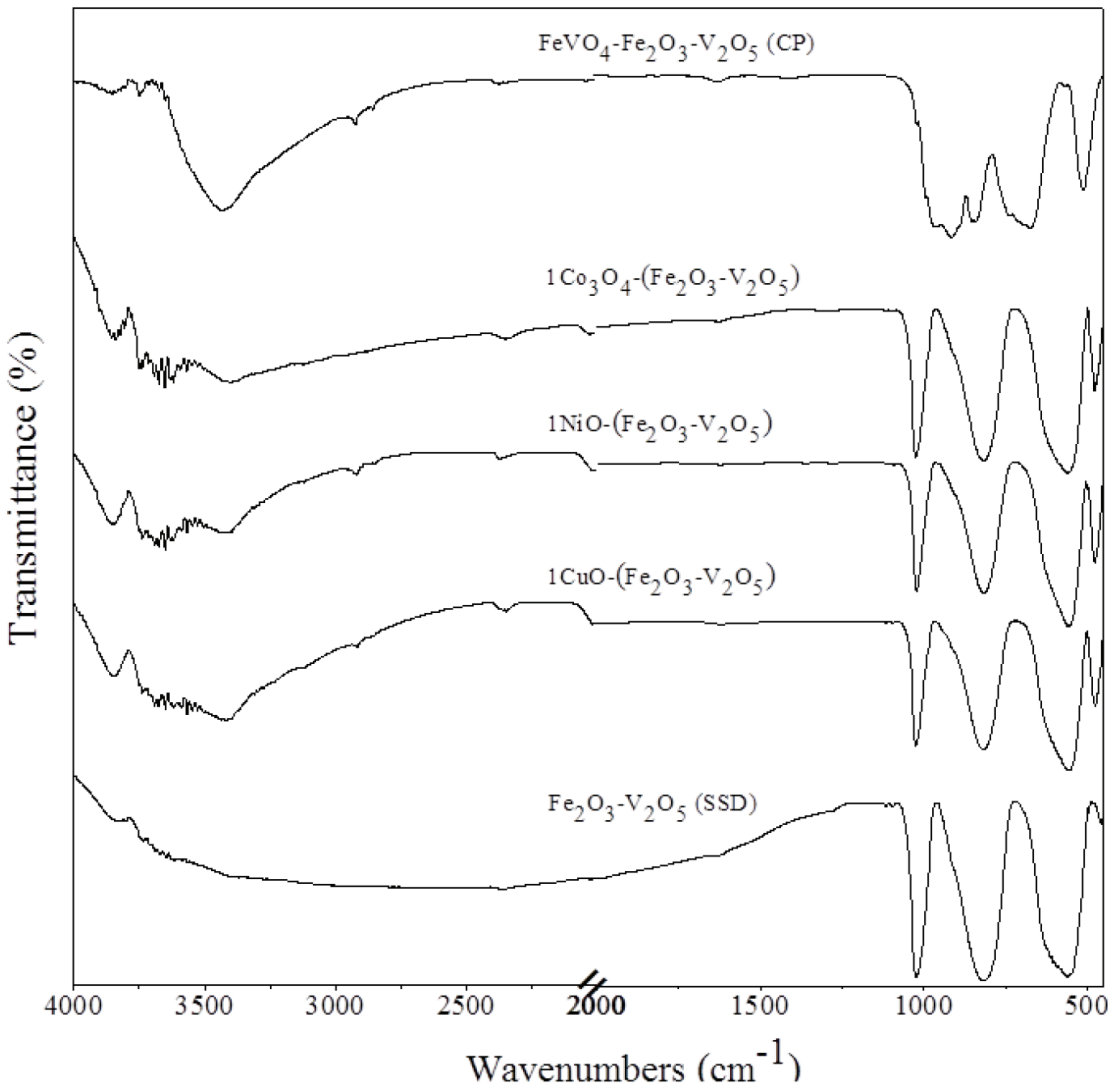
FTIR spectra of V2O5, Fe2O3, Fe2O3 – V2O5 (SSD), Fe2O3 – V2O5 (CP), and metal-doped Fe2O3 – V2O5 catalysts.

FTIR spectra analysis was performed to investigate the structure and functional groups of the materials, as exhibited in Figure 4. All of FeV-Pure samples exhibit almost the same FT-IR spectra, showing the structures of Fe_2_O_3_ and V_2_O_5_ did not change after metal oxide addition. Furthermore, light shifts occurred in the fingerprint regions. The sliding structure is known to be caused by strong interaction between FeV-Pure and metal oxides [46]. However, the vibration spectras of the metal oxides are not significant due to the low amount (1 wt%) of metal oxide additions. The presence of bands around 1000–1030 cm−1 represents V-O (vanadyl oxygen) stretching mode of vibration [47] and the bands around 735–825 cm−1 are also due to V-O-V stretching modes are viewed for nanocomposites [48]. The peaks at 470 cm−1, 555 cm−1, and 680 cm−1 attributed to Fe-O bond vibration of Fe_2_O_3_ [49]. It is also found that the strong and broad band at about 954 cm−1 and 911 cm−1 are terminal V-O stretching; 694 cm−1, 673 cm−1 are mixed bridging V-O-Fe stretching for FeV-Pure (CP) [50]. The results attained from FT-IR analysis further confirm the formation of mixed oxide catalysts and hence confirm the results reported by XRD analysis.

### 3.6. X-ray photoelectron spectroscopy

**Figure 5:**
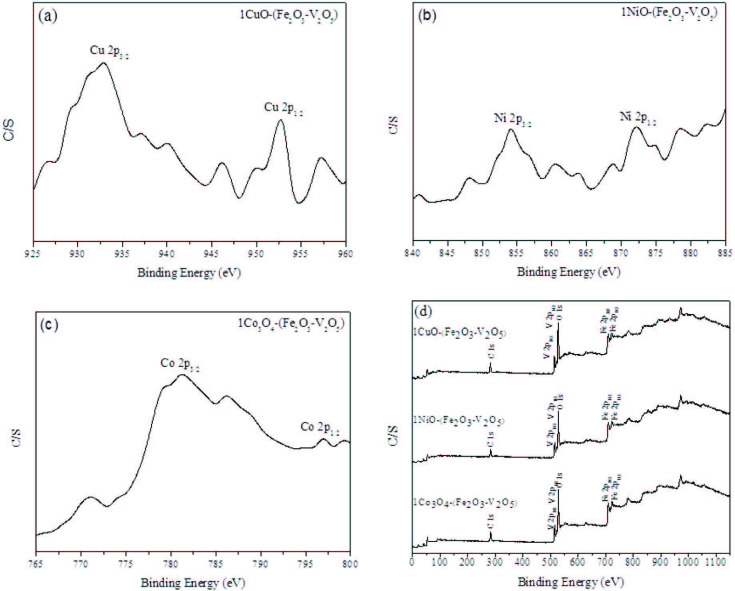
(a) XPS spectra of Cu (2p) for 1CuO – (Fe2O3 – V2O5) catalyst, (b) XPS spectra of Ni (2p) for 1NiO – (Fe2O3 – V2O5) catalyst, (c) XPS spectra of Co (2p) for 1Co3O4 – (Fe2O3 – V2O5) catalyst, (d) XPS spectra of V 2p, Fe 2p and O 1s for metal oxide added samples.

The chemical composition of the samples such as Cu, Ni and Co added Fe_2_O_3_ – V_2_O_5_ at the surface was carried out by XPS measurements. The results of XPS studies are explained in Figures 5a–5d. As shown in Figure 5a, Cu 2p core level spectrum displays two peaks with binding energies of 933 and 753 eV, which can be allocated to Cu 2p3/2 and Cu 2p1/2, indicating the presence of Cu2+. Figure 5b shows the high-resolution XPS spectrum of Ni- added FeV-Pure sample discloses Ni 2p3/2 and Ni 2p1/2 peaks at 854 and 872 eV, respectively. Furthermore, the XPS intensive peaks of Co 2p at 781 and 797 eV are resemble to Co 2p3/2 and Co 2p1/2 of Co+ ions respectively as shown in Figure 5c. Thus in accordance with the reports in the literature [51-53] and XPS results, it can be concluded that Cu, Ni and Co species are present on Fe_2_O_3_ – V_2_O_5_ sample surface, separately.

The XPS wide scan spectra recorded for metal oxide added FeV-Pure catalysts are shown in Figure 5d. It can be noticed that V 2p peaks were found besides to Fe 2p and O 1s peaks for the mixed oxide catalysts. The C 1s peak (283.3 eV) can be detected for all the powder samples due to carbon contamination. 

### 3.7. Photoluminescence spectroscopy

**Figure 6:**
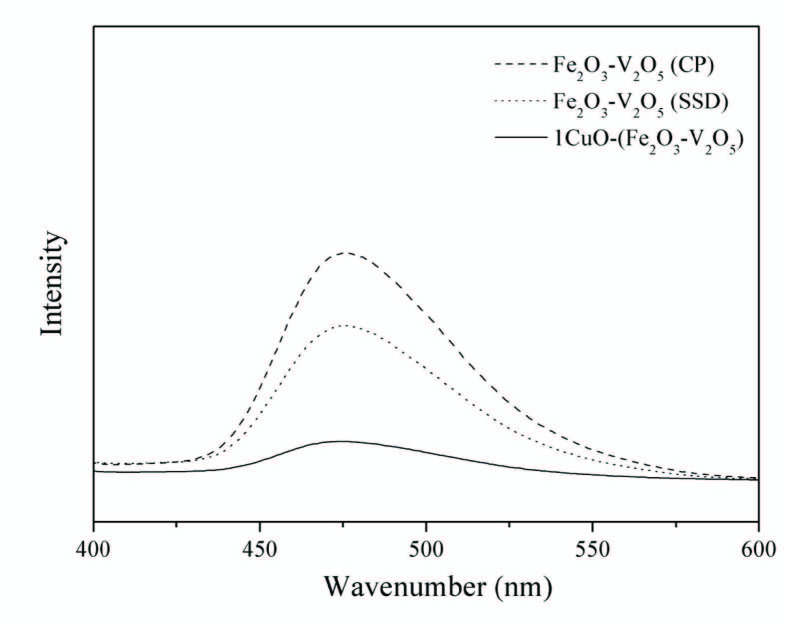
Photoluminescence spectrum of 1CuO – (Fe2O3-V2O5), Fe2O3-V2O5 (SSD) and Fe2O3-V2O5 (CP) samples.

Figure 6 indicates the photoluminescence (PL) spectra of 1CuO-(FeV-Pure), FeV-Pure, and FeV-Pure (CP). PL emission spectra is beneficial to demonstrate the efficacy of charge carrier entrapping, emigration, and transposition, and also to comprehend the fortune of electron–hole pairs in semiconductor grains because PL emanation arises as of the separation and recombination of free charge carriers [54]. When the intensity of PL becomes low, it implies the diminishing of recombination and hence possibly higher photocatalytic efficiency [55]. As shown in Figure 6, all samples exhibited PL emission spectra at peak around 475 nm at different intensities. It is possible to see from the PL spectra that the relative intensity of the emission spectra of 1CuO-(FeV-Pure) is at its lowest, indicating that recombination rates of electrons and holes are low. This suggests that CuO addition is useful to hinder the recombination of electrons and holes and thus enhance the photocatalytic activity. These findings are required features that are expected from the metal oxide additions. 

### 3.8. Photocatalytic activity

The kinetics of photocatalytic degradation of numerous organic compounds has usually been related with the Langmuir–Hinshelwood equation. Therefore, the photocatalytic reaction is the rate control step. This equation associates the degradation with the concentration of organic molecules according to Equation 1 [56]. 

r = –dC/dt = krKadsC/(1 + KadsC) (Eq.1)

where r is for the initial reaction rate (mg L–1 min–1), kr is for the Langmuir-Hinshelwood reaction rate constant (mg L–1 min–1) and K is for the Langmuir adsorption constant (L mg–1).

Photocatalysis of a heterogeneous system comprises many steps, like adsorption, diffusion, and reaction; apposite dispersal of pores is advantageous for the diffusion of reactants and products that enhances the photocatalytic reaction [57]. Dark reactions were also performed before the photocatalytic reaction to ensure adsorption/desorption equilibrium in the existence of catalyst with no irradiation.

Effects of different light sources (UV-A, UV-B, and daylight) on the photodegradation of 2,4-DCP over FeV-Pure catalyst were studied. Under UV-A illumination, 2,4-DCP was more effectively degraded to its by-products and carboxylic acids. It is clearly seen that the complete degradation (%100) of 2,4-DCP could be achieved in 120 min under UV-A irradiation, while only %70 conversion was reached under UV-B illumination. FeV-Pure sample exhibited a lower efficiency (57%) in degradation of 2,4-DCP under daylight irradiation.

Photocatalytic experiments were carried out at the following conditions: (i) degradation of 2,4-DCP using UV-A light in the absence of Fe_2_O_3_-V_2_O_5_, (ii) degradation of 2,4-DCP with FeV-Pure in dark, and (iii) degradation of 2,4-DCP using UV-A light in the existence of Fe_2_O_3_-V_2_O_5_. The results of the studies showed that direct photolysis could not trigger any considerable degradation using UV-A irradiation. Furthermore, some loss was seen due to the adsorption of 2,4-DCP on the surface of FeV-Pure in the existence of FeV-Pure with no irradiation. The adsorption caused 25% degradation in 60 min whereas the 17% degradation in 60 min occurred with the photolysis reaction. The irradiation using UV-A light in the existence of catalyst brought about 100% degradation of 2,4-DCP in 120 min. 

**Figure 7:**
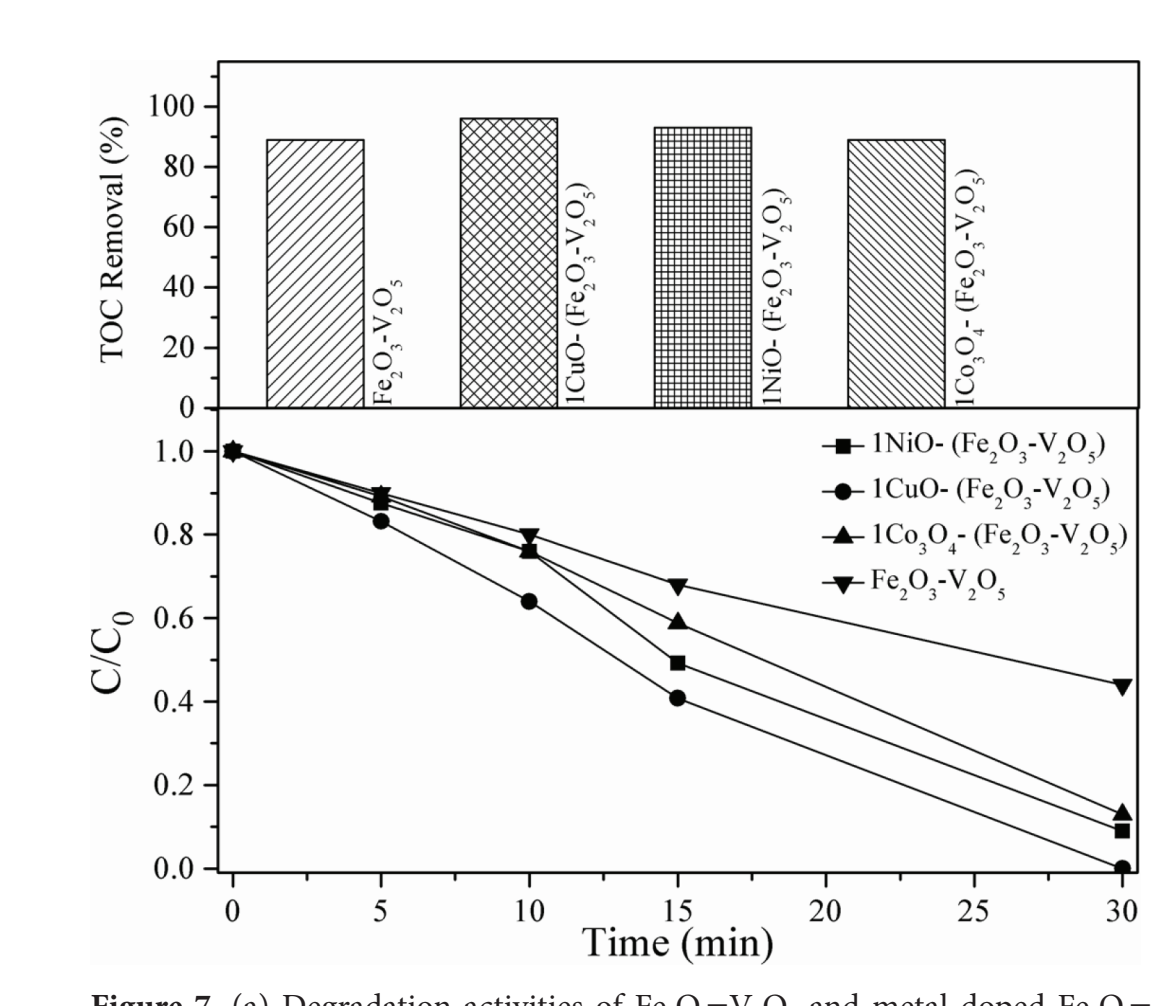
(a) Degradation activities of Fe2O3 – V2O5 and metal-doped Fe2O3 – V2O5 catalysts, (b) TOC removal for photocatalytic degradation of 2,4-DCP.

Photocatalytic efficiencies of the catalysts were studied through the degradation of 2,4-DCP in an aqueous solution in the presence of a small amount of H_2_O_2_ under UV-A illumination. The results are presented in Figure 7a and Table. All the metal-oxide-doped catalysts exhibited good photocatalytic properties. Among these catalysts, 1CuO – (FeV-Pure) catalyst showed the maximum of 100% degradation of 2,4-DCP (the highest activity) within 30 min. In addition, we performed 1CuO – (FeV-Pure) catalyst with visible light irradiation. However, it showed 75% degradation.

Satheesh et al. [58] investigated the degradation of acid red-27 dye for transition metal (M = Cu, Ni and Co)-doped iron oxide (Fe_2_O_3_) nanoparticles under visible light radiation. The photo-degradation results revealed that Cu–Fe_2_O_3_ is a better photocatalyst. The highest activity explained that the photocatalytic efficiency of Cu–Fe_2_O_3_ is influenced by the surface area, particle size and optical property.

Similarly, Park et al. [59] reported the gas-phase CO oxidation performance of doped and codoped transition metals such as Co, Cu, and Ni into CeO_2_ by temperature-programmed reaction mass spectrometry.  It was observed that Cu-containing samples exhibited greater CO performance whereas Ni-, Co-, and CoNi-doped samples exhibited low performances. This research offers profounder information of surface treatment effects that may be helpful in developing effective catalysts.

The performance of CuO – FeV-Pure catalysts with a series of different CuO loadings (0.5, 1, 3, 5, 7 wt%) was also studied to comprehend the effect of doping of the catalytic activity of binary metal oxide catalyst. According to these results, the catalyst added up to 1 wt% showed the optimal catalytic performance for 2,4-DCP degradation (100%). With the copper oxide addition from 0.5 to 1 wt% CuO, 2,4-DCP degradation rate increases first, decreasing when the amount of copper is high (3, 5, 7 wt%). This is due to the fact that Cu(II) turns into the recombination cores of the photo-induced electrons and holes, which hinders the photocatalytic reactions. In addition to this result, a decrease in activity can be described by partial blocking of the active species of FeV-Pure due to the creation of bigger CuO particles at higher CuO additions. 

Yu and Ran [60] examined the photocatalytic activities of Cu(OH)2 clusters-modified TiO_2_ (P25 Degussa) for hydrogen production. The deposition of Cu(OH)2 clusters on the surface of TiO_2_ was produced at different mol % ratios (0, 0.05, 0.25, 1, 2, and 7) by a facile precipitation approach. The highest H2-fabrication rate was observed with 0.29 mol Cu(OH)2 addition. This was attributed to the fact that small Cu(OH)2 clusters was evenly distributed on the surface of the TiO_2_ nanoparticles. In addition, the catalytic activity of 1 wt% CuO to FeV-Pure was higher than those of FeV-Pure (CP), V_2_O_5_, and Fe_2_O_3_ samples. These results imply that the doping of transition metal oxide and binary oxide structure play an important role on the photocatalytic activity.

In addition, the reusability of the 1CuO – (FeV-Pure) catalyst was studied on fresh dye samples (5 trials). 1CuO – (FeV-Pure), when used for the ﬁrst time, could degrade 98.45% 2,4-DCP, with a small change (to 94.21%) in the efﬁciency when used for five times. This decrease in the efﬁciency for 1CuO – (FeV-Pure) catalyst resulted probably from the photocorrosion effect.

The catalyst synthesized using the CP method exhibited low activity. The conversion of 2,4-DCP reached 48% at the completion of 30 min using the FeV-Pure (CP). This result demonstrated that the fabrication method substantially affected the interface between Fe_2_O_3_ and V_2_O_5_. FeV-Pure prepared by the solid state dispersion method displays the mutual chemical interaction between Fe_2_O_3_ and V_2_O_5_, which leads to the fastest interfacial charge transfer and the most influential partition of photo-generated electron–hole pairs, and hence, exhibits the highest photocatalytic activity. Moreover, agglomeration is observed in the SEM images of catalyst prepared using the CP method. The morphology of the catalyst prepared using the SSD method is different from that of the catalyst prepared using the CP. The primary particles show irregular sizes and shapes. The distribution of particle size appears to be wider than those of the other samples. Based on the SEM images of catalysts, CuO cluster is more effective in photodegradation reactions.

Sun et al. [61] investigated the effect of impregnation and mechanical mixing methods for pyrolysis of biomass experiments with Fe/CaO catalysts. Based on the results of structure characteristics and catalytic activities, it was concluded that the higher activity of im-Fe/CaO catalyst is ascribed to the stronger metal support interaction between Fe and CaO.

Figure 7b shows the TOC elimination results on the photocatalytic degradation of 2,4-DCP with 1CuO – (FeV-Pure), 1NiO – (FeV-Pure), 1CO_3_O_4_ – (FeV-Pure), and FeV-Pure. In 30 min, 25 ppm 2,4-DCP can be totally degraded by 1CuO – (Fe_2_O_3_ – V_2_O_5_), and 96% TOC elimination can be attained in 30 min. This result emphasizes the accomplishment of the total mineralization. 

From the photocatalytic results, the improved photocatalytic efficiency of 1CuO – (FeV-Pure) could be ascribed to apposite dispersal of pores and high separation rate of photo-induced charge carriers. Furthermore, the decrease in the particle size due to Cu2+ addition is the reason for higher efficiency of the catalyst. The crystal structure, crystallinity, band gap, synthesis method, morphology, particle shape, and surface area of a material are the significant elements influencing its photocatalytic activity [62-64]. However, these are not the only factors impacting high reactivity for the degradation of 2,4-DCP. The reaction with the metal-doped binary metal oxide catalyst is more essential. The optimum photo-degradation activity was seen with 1CuO wt% FeV-Pure. 

Neppolian et al. [65] explained that the electron injection was shown to be the main factor in the high activity of the binary metal oxide catalysts beside other physicochemical properties. The transfer of electrons from ZrO_2_ to TiO_2_ was seen to be the major phenomenon in the binary metal oxide catalyst in the course of the chemical interactions between ZrO_2_ and TiO_2_ in the form of Ti-O-Zr- bond. Furthermore, Wu et al. [66] have expressed the creation of a joint chemical interaction between the pure oxides when they are coprecipitated together (-Ti-O-Zr-) resulting in the enhancement of the photocatalytic properties. 

**Figure 8:**
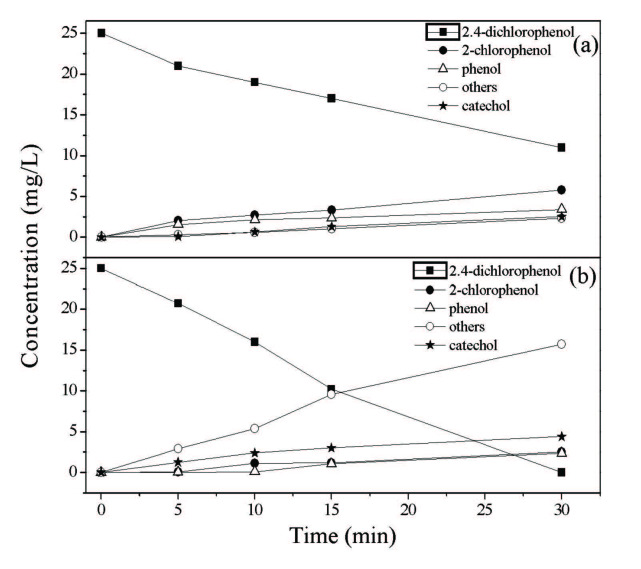
Concentration profiles for intermediates obtained during the photocatalytic oxidation reaction using (a) Fe2O3 – V2O5, (b) 1CuO – (Fe2O3 – V2O5).

Concentration profiles for 2,4-DCP, 2-chlorophenol, phenol, catechol, and others such as ring-opening products are gained in the photocatalytic oxidation reaction via FeV-Pure and 1CuO – (FeV-Pure) are shown in Figure 8. In addition, the dispersal of intermediates was similarly compared. According to HPLC results, 2-chlorophenol, phenol, and catechol were distinguished as key intermediates by making use of FeV-Pure (Figure 8a). In addition to these products, ring-opening products (others) were also noticed at very low concentrations. However, 2-chlorophenol, phenol, and catechol were found to be at very low concentrations for 1CuO – (FeV-Pure) catalyst (Figure 8b). Moreover, it was observed that ring-opening products (others) were found to be at higher concentrations by means of metal oxide added 1CuO – (FeV-Pure) catalyst compared to FeV-Pure. These intermediates undertake additional photocatalytic oxidation to ring cleavage and to produce carboxylic acids and aldehydes which provide CO_2_ and H_2_O because of decarboxylation.

As known, the chemical nature and some structural features of a catalyst significantly affect its photocatalytic properties. These results confirm that 1CuO – (FeV-Pure) is more effective when compared to other catalysts. The findings of this work also indicate that the photochemical reaction has a significant influence on the degradation of 2,4-DCP under the stated conditions.

## 4. Conclusion

FeV-Pure binary metal oxide catalysts were successfully synthesized using the SSD and CP methods. The catalysts show higher photocatalytic efficiency in degrading 2,4-DCP under the application of UV-A irradiation. The chemical interaction between Fe_2_O_3_ and V_2_O_5_ expressed as Fe-O-V-bond may affect the photo-efficiency of binary oxide catalysts.

Additionally, the effects of copper, nickel, and cobalt oxide additions on the photocatalytic degradation of 2,4-DCP were investigated. The presence of metal oxides over semiconductor (FeV-Pure) surface was determined using XPS measurements. 1CuO wt% FeV-Pure revealed the highest percentage of 2,4-DCP degradation (100%) and the highest reaction rate (1.83 mg L−1 min−1) in 30 min. It is noteworthy to mention that structural, morphological, optical, and photocatalytic properties of the catalysts provide substantial contribution throughout the photo-catalytic process.

The outcomes of this work imply that using the 1CuO – (FeV-Pure) in the photocatalytic degradation of phenolic compounds enhances the degradability of the wastewater containing chlorophenols. The development of such heterostructured nanocomposites could lead to a new perception that may initiate the production of more efficient catalysts for photocatalytic degradation of organic materials as well as a potential industrial application. 

## References

[ref1] (2013). Adsorption and photocatalytic degradation of 2,4-dichlorophenol in TiO_2_ suspensions. Effect of hydrogen peroxide, sodium peroxodisulphate and ozone. Applied Catalysis A: General.

[ref2] (2014). Assemblies of nanoparticles of CeO_2_–ZnTi-LDHs and their derived mixed oxides as novel photocatalytic systems for phenol degradation. Applied Catalysis B: Environmental.

[ref3] (2014). Competitive role of structural properties of titania–silica mixed oxides and a mechanistic study of the photocatalytic degradation of phenol. Applied Catalysis B: Environmental.

[ref4] (2012). -dichlorophenol contaminated water by visible light-enhanced WO_3_ photoelectrocatalysis. Remediation of 2.

[ref5] (2013). Hong SW et al. Chemical Engineering Journal.

[ref6] (2012). Complete dechlorination of pentachlorophenol by a heterogenous SiO_2_–Fe–porphyrin catalyst. Applied Catalysis B: Environmental.

[ref7] (2004). Degradation of chlorophenols by means of advanced oxidation processes: a general review. Applied Catalysis B: Environmental.

[ref8] (2015). Transition metal loaded TiO_2_ for phenol photo-degradation..

[ref9] (2016). Photocatalytic reductive degradation of polybrominated diphenyl ethers on CuO/TiO_2_ nanocomposites: a mechanism based on the switching of photocatalytic reduction potential being controlled by the valence state of copper.

[ref10] (2015). Synthesis of α-Fe_2_O_3_/ZnO composites for photocatalytic degradation of pentachlorophenol under UV–vis light irradiation. Ceramic International.

[ref11] (2015). Visible-light-induced photocatalytic performances of ZnO–CuO nanocomposites for degradation of 2,4-dichlorophenol. Chinese Journal Catalysis.

[ref12] (2012). Photocatalytic activity of AgI sensitized ZnO nanoparticles under visible light irradiation. Powder Technology.

[ref13] (2013). Visible light assisted photo catalytic activity of TiO_2_-metal vanadate (M = Sr, Ag and Cd) nanocomposites. Materials Science in Semiconductor Processing.

[ref14] (2016). Morphology engineering of V_2_O_5_/TiO_2_ nanocomposites with enhanced visible light-driven photofunctions for arsenic removal. Applied Catalysis B: Environmental.

[ref15] (2014). Enhanced photocatalytic activity of V_2_O_5_–ZnO composites for the mineralization of nitrophenols. Chemosphere.

[ref16] (2016). Photocatalytic properties of Au/Fe_2_O_3_ nano-composites prepared by co-precipitation. Advanced Powder Technology.

[ref17] (2015). Facile synthesis and high activity of novel BiVO_4_/FeVO_4_ heterojunction photocatalyst for degradation of metronidazole. Applied Surface Science.

[ref18] (2012). Catalytic destruction of pentachlorobenzene in simulated flue gas by a V_2_O_5_–WO_3_/TiO_2_ catalyst. Chemosphere.

[ref19] (2013). Hydrogen production by glycerol reforming in supercritical water over Ni/MgO-ZrO_2_ catalyst. Journal of Energy Chemistry.

[ref20] (2015). Enhanced magnetic property and photocatalytic activity of UV-light responsive N-doped Fe_2_O_3_/FeVO_4_ heterojunction. Ceramics International.

[ref21] (2005). Visible light active platinum-ion-doped TiO_2_ photocatalyst. The Journal of Physical Chemistry B.

[ref22] (2006). Transition metal-doped titanium(IV) dioxide: characterisation and influence on photodegradation of poly(vinyl chloride). Polymer Degradation and Stability.

[ref23] (2004). Effects of sol–gel procedures on the photocatalysis of Cu/TiO_2_ in CO_2_ photoreduction. Journal of Catalysis.

[ref24] (2004). Characterization of Fe-TiO_2_ photocatalysts synthesized by hydrothermal method and their photocatalytic reactivity for degradation of XRG dye diluted in water. Journal of Molecular Catalysis A: Chemical.

[ref25] (2010). Iron-coated TiO_2_ nanotubes and their photocatalytic performance. Journal of Material Chemistry.

[ref26] (2008). Hydrothermally stabilized Fe(III) doped titania active under visible light for water splitting reaction. International Journal of Hydrogen Energy.

[ref27] (2006). Effects of Fe-doping on the photocatalytic activity of mesoporous TiO_2_ powders prepared by an ultrasonic method. Journal of Hazardous Materials.

[ref28] (2004). Structural and spectroscopic studies of iron (III) doped titania powders prepared by sol-gel synthesis and hydrothermal processing. Dyes and Pigments.

[ref29] (2013). Transition metal coated TiO_2_ nanoparticles: synthesis, characterization and their photocatalytic activity. Applied Catalysis B: Environmental.

[ref30] (2007). -Fe_2_O_3_ composite photocatalyst activate in the degradation of 4-chlorophenol and orange II under daylight irradiation. Applied Catalysis B: Environmental.

[ref31] (2015). Synthesis of mesoporous α-Fe_2_O_3_ via sol–gel methods using cellulose nano-crystals (CNC) as template and its photo-catalytic properties. Materials Letters.

[ref32] (2002). Influence of V_2_O_5_ content on ammoxidation of 3-picoline over V_2_O_5_/AlF3 catalysts. Catalysis Communications.

[ref33] (2010). Novel counter electrode V_2_O_5_/Al for solid dye-sensitized solar cells. ACS Applied Materials Interfaces.

[ref34] (2010). Self assembled V_2_O_5_ nanorods for gas sensors. Current Applied Physics.

[ref35] (1993). Fabrication and characterization of amorphous lithium electrolyte thin films and rechargeable thin-film batteries. Journal of Power Sources.

[ref36] (2012). Porous V_2_O_5_ micro/nano-tubes: synthesis via a CVD route, single-tube-based humidity sensor and improved Li-ion storage properties. Journal of Materials Chemistry.

[ref37] (2011). One pot synthesis of self-assembled V_2_O_5_ nanobelt membrane via capsule-like hydrated precursor as improved cathode for Li-ion battery. Journal of Materials Chemistry.

[ref38] (2014). Doping of Co into V_2_O_5_ nanoparticles enhances photodegradation of methylene blue. Journal of Alloys and Compounds.

[ref39] (2008). Ternary metal oxide catalysts for selective oxidation of benzene to phenol. Journal of Industrial and Engineering Chemistry.

[ref40] (2010). Oxidative dehydrogenation of butane over substoichiometric magnesium vanadate catalysts prepared by citrate route. Journal of Non-Crystalline Solids.

[ref41] (2009). Andersson A. Stability and performance of supported Fe–V- oxide catalysts in methanol oxidation. Journal of Catalysis.

[ref42] (2010). The impact of silver modification on the catalytic activity of iodine-doped titania for p-chlorophenol degradation under visible-light irradiation. Journal of Molecular Catalalysis A: Chemical.

[ref43] (2010). Metal oxide gas sensors: sensitivity and influencing factors. Sensors.

[ref44] (2013). CoFe2O_4_/TiO_2_ nanocatalysts for the photocatalytic degradation of reactive red 120 in aqueous solutions in the presence and absence of electron acceptors. Chemical Engineering Journal.

[ref45] (2013). Photocatalytic degradation of phenol and benzoic acid using zinc oxide powders prepared by the sol–gel process. Alexandria Engineering Journal.

[ref46] (2002). Hydrothermal synthesis and optical property of nano-sized CoAl_2_O_4_ pigment. Materials Letters.

[ref47] (2008). Mixed amorphous and nanocrystalline TiO_2_ powders prepared by sol–gel method: characterization and photocatalytic study. Materials Chemistry and Physics.

[ref48] (2004). Vibrational spectra of alumina- and silica-supported vanadia revisited: an experimental and theoretical model catalyst study. Journal of Catalysis.

[ref49] (2009). Structural investigation of MFe2O_4_ (M) Fe, Co) magnetic fluids. Journal of Physical Chemistry C.

[ref50] (2015). Synthesis of surfactant-assisted FeVO_4_ nanostructure: characterization and photocatalytic degradation of phenol. Journal of Molecular Catalalysis A: Chemical.

[ref51] (2016). Scalable synthesis of cubic Cu1.4S nanoparticles with long-term stability by laser ablation of salt powder. Chemical Communications.

[ref52] (2015). Single-crystalline Ni(OH)2 nanosheets vertically aligned on a three-dimensional nanoporous metal for high-performance asymmetric supercapacitors. Journal of Materials Chemistry A.

[ref53] (2016). Self-assembly of ultrathin mesoporous CoMoO_4_ nanosheet networks on flexible carbon fabric as a binder-free anode for lithium-ion batteries. New Journal of Chemistry.

[ref54] (2000). Variation of langmuir adsorption constant determined for TiO_2_-photocatalyzed degradation of acetophenone under different light intensity. Journal of Photochemistry and Photobiology A: Chemistry.

[ref55] (2007). An efficient bicomponent TiO_2_/SnO_2_ nanofiber photocatalyst fabricated by electrospinning with a side-by-side dual spinneret method. Nano Letters.

[ref56] (2000). Variation of langmuir adsorption constant determined for TiO_2_ photocatalyzed degradation of acetophenone under different light intensity. Journal of Photochemistry and Photobiology A: Chemistry.

[ref57] (2012). Improved photocatalytic performance of ZnO prepared by sol–gel method with the assistance of CTAB. Materials Letters.

[ref58] (2014). Visible light responsive photocatalytic applications of transition metal (M = Cu, Ni and Co) doped α-Fe_2_O_3_ nanoparticles. Journal of Environmental Chemical Engineering.

[ref59] (2014). Surface treatment effects on CO oxidation reactions over Co, Cu, and Ni-doped and codoped CeO_2_ catalysts. Chemical EngineeringJournal.

[ref60] (2011). Facile preparation and enhanced photocatalytic H2-production activity of Cu(OH)2 cluster modified TiO_2_. Energy and Environmental Science.

[ref61] (2015). Effect of preparation method on structure characteristics and fast pyrolysis of biomass with Fe/CaO catalysts. Journal of  Analytical and Applied Pyrolysis.

[ref62] (2006). Effects of structural variation on the photocatalytic performance of hydrothermally synthesized BiVO_4_. Advanced Functional Materials.

[ref63] (2007). Shape, size and photocatalytic activity control of TiO_2_ nanoparticles with surfactants. Journal of Photochemistry and Photobiology A: Chemistry.

[ref64] (2009). Adsorption and photocatalytic degradation of phenol and 2,4 dichlorophenoxiacetic acid by Mg–Zn–Al layered double hydroxides. Applied Catalysis B Environmental.

[ref65] (2007). Synthesis and characterization of ZrO_2_–TiO_2_ binary oxide semiconductor nanoparticles: application and interparticle electron transfer process. Applied Catalysis A General.

[ref66] (1984). Nonoxidative dehydrogenation of ethylbenzene over TiO_2_-ZrO_2_ catalysts: II. The effect of pretreatment on surface properties and catalytic activities. Journal of Catalysis.

